# Cellulose Acetate Fabrics Loaded with Rhodamine B Hydrazide for Optical Detection of Cu(II)

**DOI:** 10.3390/molecules25163751

**Published:** 2020-08-17

**Authors:** Rania E. Morsi, Moataz Elsawy, Ilse Manet, Barbara Ventura

**Affiliations:** 1 Egyptian Petroleum Research Institute, Nasr City, Cairo 11727, Egypt; mo3taz_elsawy@yahoo.com; 2 Istituto per la Sintesi Organica e la Fotoreattività (ISOF), Consiglio Nazionale delle Ricerche (CNR), 40129 Bologna, Italy; ilse.manet@isof.cnr.it

**Keywords:** cellulose acetate, rhodamine B hydrazide, optical sensing, membrane, fibers

## Abstract

In this work, different materials were fabricated from cellulose acetate, loaded with rhodamine B hydrazide and tested as Cu(II) optical sensor. We prepared membranes displaying a sub-micron porous structure using the phase inversion technique, clusters of fibers with varying diameter depending on the preparation procedure using electrospinning, and casted films presenting a smooth non porous structure. Loading of rhodamine B hydrazide on the fabrics after their production was found to be the best procedure to ensure the stability of the dye in the polymeric materials. Absorption and emission analysis of the solid substrates revealed the presence of the dye on the porous fabrics and allowed to choose the most suited materials and loading conditions to test their response towards Cu(II) ions. Reaction of the loaded rhodamine B hydrazide with Cu(II) was confirmed by absorption and emission spectroscopies and by confocal fluorescence imaging, through detection of the product rhodamine B. The results point to promising sensing applications of the prepared composite materials.

## 1. Introduction

Fast, practical, and reliable detection of contaminants is a target of growing interest, as a result of the increased awareness and the enhanced levels of safety standards related to human health and the environment.

An important motivation is the growing number of diseases related to metal contaminated water. For example, long exposure to high copper concentrations can be toxic to living organisms and aqua-biological systems [1], thus, the development of efficient strategies for Cu(II) detection in water and environmental samples is a stringent challenge. Colorimetric or fluorimetric sensors are optical active dyes which show modifications of their absorption or fluorescence features upon interaction with the targeted species. Among these dyes, xanthenes, a class of photoactive materials, are widely used for targeting Cu(II), according to the ring opening mechanism induced by the presence of the metal ion, where the colorless and weakly fluorescent spiro form transforms into the open molecule, pink in color, and strongly fluorescent [2,3,4].

Immobilization of such molecules on supporting solid fabrics can facilitate and widen their range of applications. In general, integration of different materials with different properties results in a possibility of gaining additional features, as being amenable for sustainable use and recycling. The sensing molecules should be immobilized onto the support fabrics in a non-leaching way, ensuring at the same time their availability for the interaction with the metal ion. Research efforts have been recently devoted to the development of suitable supports and the optimization of immobilization techniques, providing potential capability for rapid, efficient, and low-cost sensing [5,6,7].

Most available supports are composed of polymeric materials fabricated with different morphological structures. Among them, porous membranes prepared by phase inversion showed several advantages and found interesting applications [8,9]. Electrospun polymeric nanofibers have also many interesting features due to their unique morphological structure, as the high porosity, the network structure with interconnected voids and the high surface area-to-volume ratio [10,11,12,13].

We previously reported the optimization of the immobilization of rhodamine B hydrazide (RBH) onto polymeric fabrics for the detection of Cu(II) ions in water through absorption and emission changes of the RBH dye that retained its optical features after being loaded into the porous matrices [14]. Polysulfone was used as scaffold material, fabricated in three different forms: casted films, porous membranes, and nanofibers prepared by solution casting, phase inversion, and electrospinning, respectively.

The aim of the current work is the development of biocompatible polymeric fabrics loading RBH for Cu(II) sensing applications using cellulose acetate, a natural polymer derivative, improving the sensory response of the loaded dye through the optimization of the fabrics morphology, and the loading procedure monitored by analysis of the dye absorption and emission properties.

## 2. Results and Discussion

### 2.1. Fabrication of Cellulose Acetate Matrices

**Scheme 1:**
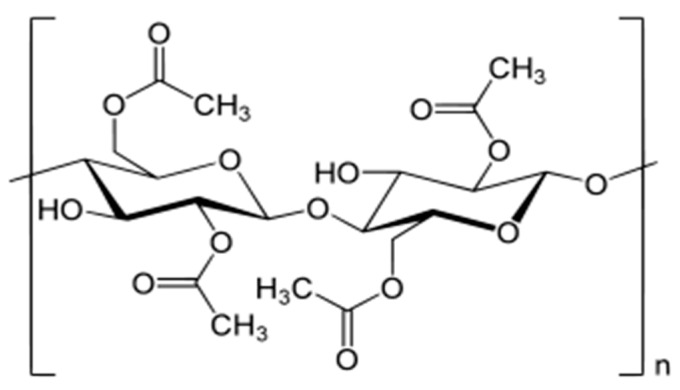
Chemical structure of cellulose acetate.

Cellulose acetate (Scheme 1) is a polymer that can be used to produce diverse types of fabrics.

**Figure 1:**
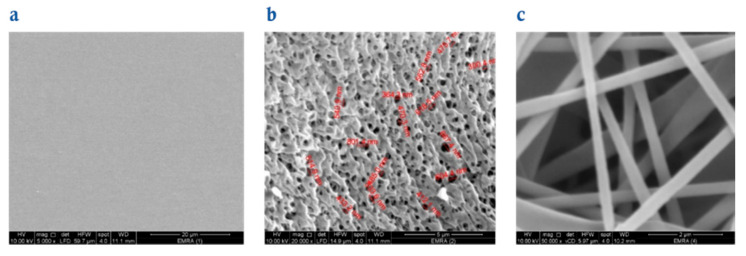
Scanning Electron Microscopy (SEM) images of the prepared cellulose acetate materials as: (**a**) casted film, (**b**) porous membrane, and (**c**) nanofibers from dimethylformamide (DMF).

**Figure 2:**
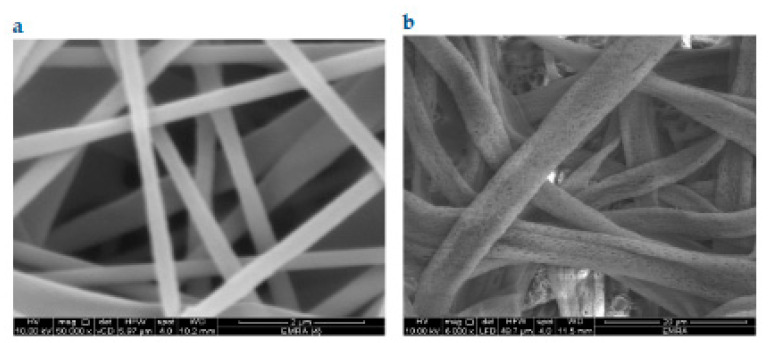
SEM images for the electrospun cellulose acetate nanofibers obtained in: (**a**) DMF and (**b**) dichloromethane (DCM)/acetone mixture.

In this work, cellulose acetate was fabricated as: (i) casted films by solution casting, (ii) porous membranes by phase inversion, and (iii) electrospun nanofibers by electrospinning technique. Morphological analysis with Scanning Electron Microscopy (SEM), showed that the three materials present very different surface features: the casted film has a smooth non-porous surface, the membrane has a porous surface with pore diameters in the sub-micron level and the electrospinning process from dimethylformamide (DMF) solution produced sub-micron fibers (Figure 1 for SEM images). Noticeably, the solvent and working conditions used in the electrospinning process greatly affect the morphology of the nanofibers [15,16,17] (Figure 2): the use of dichloromethane (DCM)/acetone mixture produced larger and flatter fibers (Figure 2b), with the macroscopic outcome of a soft fabric with cotton wool like texture.

### 2.2. Loading of RBH into the Cellulose Acetate Fabrics: Morphological and Spectroscopic Characterization

**Scheme 2:**
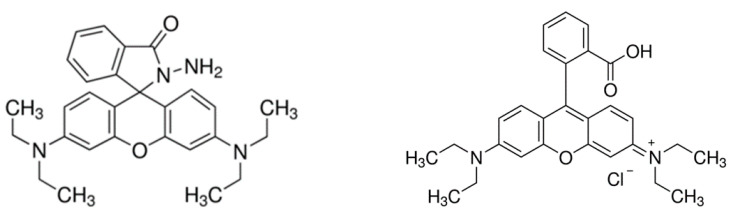
Structure of Rhodamine B hydrazide (RBH, left) and Rhodamine B (RB, right).

The chemical structures of RBH and its open form RB are shown in Scheme 2.

Ethanol was chosen as a solvent for RBH since it is well absorbed into the cellulose acetate fabrics without solubilizing the polymer.

A preliminary spectroscopic characterization of RBH and its open form RB in ethanol has been performed. Absorption and emission features of the two forms, reported in Figures S1 and S2, were found to be similar to those observed in acetonitrile [14]. The main differences reside in a red-shifted emission (maximum at 560 vs. 524 nm) for RBH and a blue shift of both absorption and emission bands (544 vs. 555 nm and 570 vs. 582 nm, respectively) for RB in ethanol with respect to acetonitrile. Excited state lifetimes of 1.6 and 3.0 ns were measured for RBH and RB, respectively [18,19,20].

RBH was loaded onto the fabrics either by pre- or post-treatment, as described in the Materials and Methods section. The structural characteristics of both the cellulose acetate and RBH, represented in Scheme 1 and Scheme 2, suggest an affinity between the dye and the polymer, given by electrostatic interactions and hydrogen bonding [21], due to the possibility of hydrophobic, hydrophilic and π–π interactions in different moieties of their structures [22].

**Figure 3:**
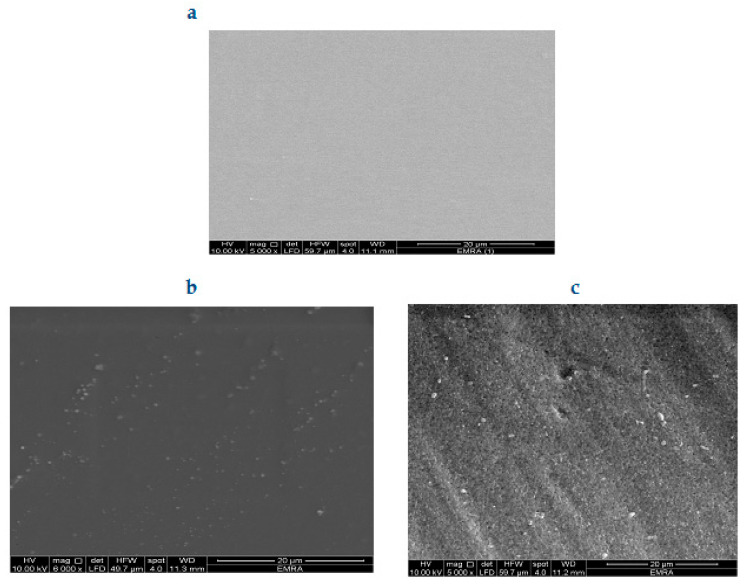
SEM images of the casted cellulose acetate film: (**a**) pure fabric, (**b**) fabric loaded with RBH by pre-treatment, and (**c**) fabric loaded with RBH by post-treatment.

**Figure 4:**
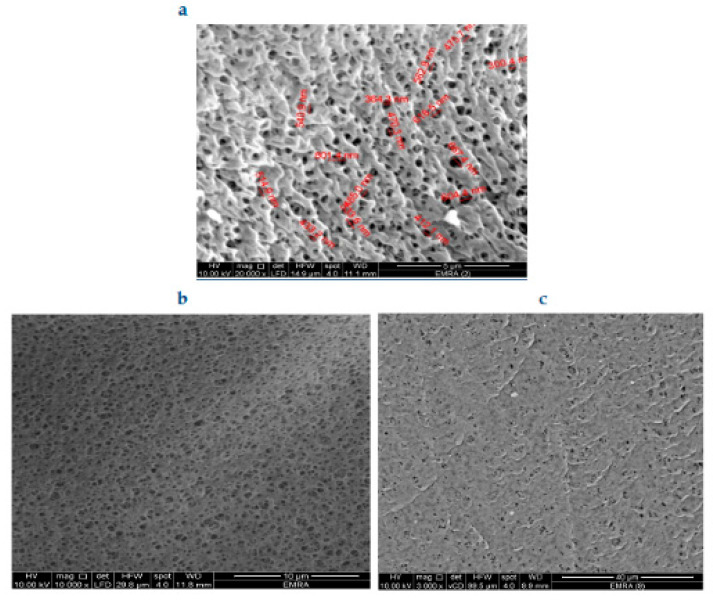
SEM images for the phase inversion deposited cellulose acetate membrane: (**a**) pure fabric, (**b**) fabric loaded with RBH by pre-treatment, and (**c**) fabric loaded with RBH by post-treatment.

**Figure 5:**
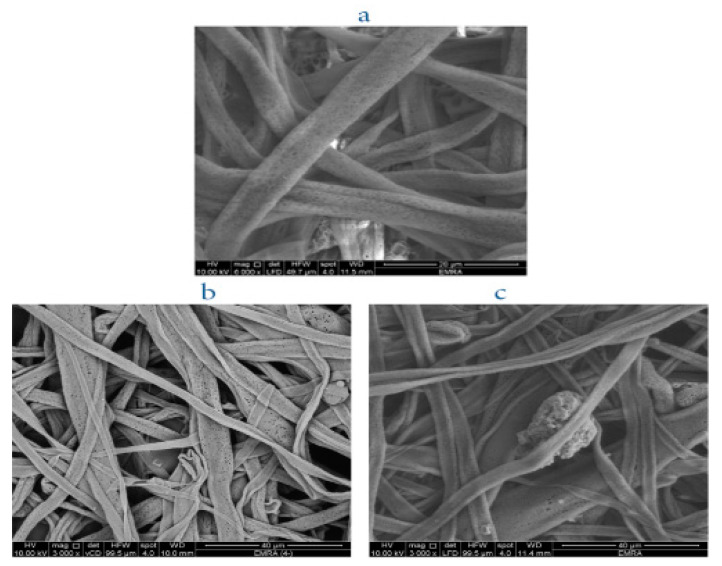
SEM images for the electrospun cellulose acetate fibers from DCM/acetone: (**a**) pure fabric, (**b**) fabric loaded with RHB by pre-treatment, and (**c**) fabric loaded with RHB by post-treatment.

From a morphological analysis of the RBH loaded materials by means of SEM (Figure 3, Figure 4 and Figure 5) it can be inferred that the loading of the dye, either by pre- or post-treatment, is not largely affecting the morphology of the materials, with the exception of a pore closure effect in the case of the phase inversion deposited membrane (Figure 4c).

In the case of RBH pre-loading in the materials, the embedded dye was found to be unstable in air, possibly due to the reaction with traces of acidity present in environmental aqueous vapor. For this reason, these materials have not been characterized from the photophysical point of view and a complete spectroscopic analysis has been performed only for the post-loaded fabrics.

**Figure 6:**
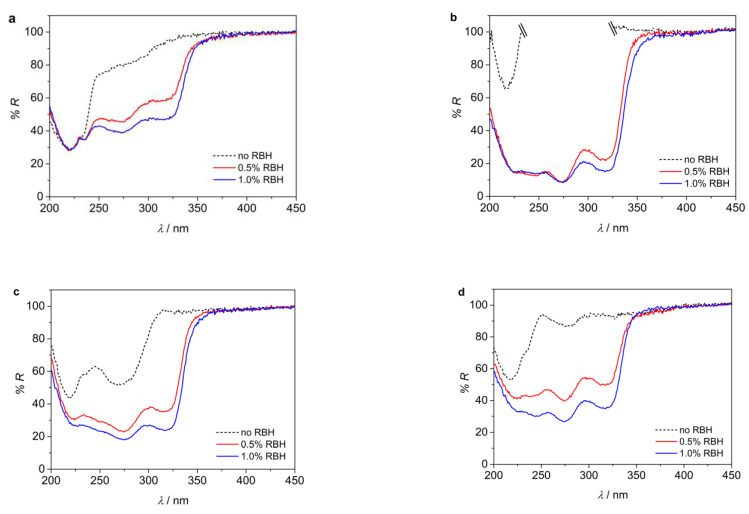
Reflectance spectra of samples with 0, 0.5, and 1% (*w*/*w*) post-loading of RBH: (**a**) casted film, (**b**) phase deposited membrane^§^, (**c**) fibers from DMF, and (**d**) fibers from DCM/acetone.

Reflectance spectra of the bare materials and of the same materials loaded with RBH by post-treatment (0.5 and 1.0% *w/w*), acquired by means of an integrating sphere, are shown in Figure 6. In all the examined samples, the spectrum of the untreated polymer consists of an envelope of bands in the region 200–350 nm. For all the treated materials, the loading of RBH was clearly confirmed by the appearance of the bands at 270 and 320 nm, typical of the RBH dye (Figure 6). In order to achieve an approximately quantitative description, the spectra have been converted in absorbance, as a function of the diffuse reflectance (f(R)), by using the Kubelka–Munk equation [23] (Figure S3). An almost quantitative doubling of RBH absorption is observed in all cases upon increasing the percentage of loading from 0.5% to 1.0% (Figure S3). The highest degree of loading is observed in the most porous material, i.e., the phase deposited membrane (Figure 6b and Figure S3b).

Emission spectra have been collected, upon excitation at 320 nm, for all samples (Figure S4). The bare polymeric material shows a weak emission in the 350–500 nm range in all cases, except for the sample of fibers obtained from DMF, where an intense and a well-defined band with maximum at 380 nm is observed (Figure S4c). Excitation spectra, collected at 430 nm, show features resembling the polymer absorption bands (Figure S5 for selected examples).

The loading of RBH results in a clear emission band at 470 nm, typical of the molecule, for porous membrane and fibers samples (Figure S4). In these samples, the emission from the polymer is suppressed and only the fluorescence from RBH is present, with a general trend towards an increased intensity for the higher degree of loading. On the contrary, the casted film loaded with RBH shows a weak emission from RBH, comparable in intensity with the faint emission of the polymer, still observable (Figure S4a). This latter result indicates a non-optimal affinity or distribution of the dye on the polymeric support, probably due to the high smoothness of the surface. It can be noticed that a shoulder (or a band) at 580 nm, typical of the open form RB, is appearing in all cases, overlapping with the RBH emission. This is due to the presence of traces of RB in the samples, easily detectable thanks to its emission properties, different from those of RBH [14]. Excitation spectra, measured at 500 and 600 nm for the porous membrane sample loaded with 1% RBH, confirmed that the band at 580 nm originates from RB (Figure S6), pointing to some degradation of the materials during the post-loading process [14].

In conclusion, the analysis allowed to ascertain that cellulose acetate is a good polymer for the loading of RBH with a post-treatment deposition, showing interesting morphological features, good stability with respect to degradation and retained optical properties of the dye. The porous samples and fibers were identified as the best materials to be used as scaffolds, and 1.0% loading has been chosen as the optimum condition.

**Figure 7:**
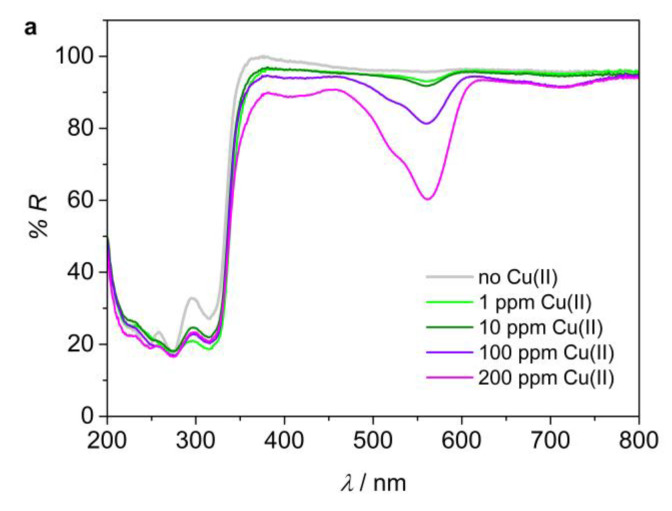

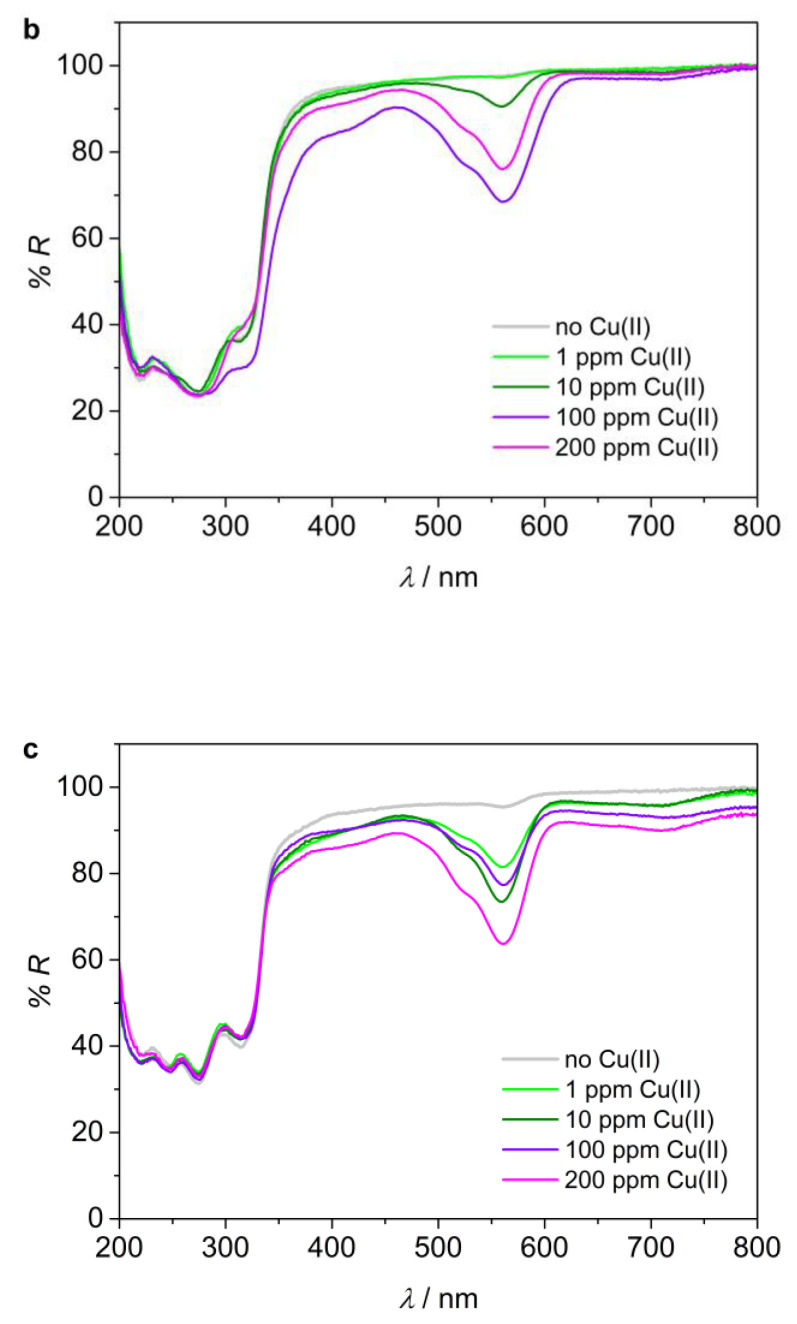
Reflectance spectra of the RBH loaded polymeric materials, prior and after 1 night immersion in CuCl_2_ water solutions (pH = 7) at different Cu(II) concentrations: (**a**) porous membrane, (**b**) fibers from DMF, and (**c**) fibers from DCM/acetone.

In order to determine the response to Cu(II) of the selected membrane and fiber samples post-loaded with RBH 1%, exposure to CuCl_2_ water solutions (pH = 7) at different Cu(II) concentrations (1–200 ppm) was performed, leaving the samples for 1 night in contact with the copper solution. Reflectance spectra of the materials, recorded after exposure, reveal in all cases the appearance of a band with maximum at 560 nm, with a general trend in its intensification upon increase of the copper concentration (Figure 7). The features of the new band lead to identify it as the main absorption band of RB, even if it is red-shifted, broader and more split with respect to the solution case (see Figure S2). These features can be ascribed to a high local concentration of the dye on the polymeric material [24]. Overall, the results testify the reaction of the deposited RBH with the Cu(II) ions present in solution, which leads to the opening of the spiro ring and formation of RB [25], and the trend in the absorption spectra points to a promising application of the prepared materials as colorimetric sensors for Cu(II). Moving towards a quantitative approach, the reflectance spectra have been converted in absorption spectra (Figure S7): absorbance values of the order of 0.1 are detected for the RB band at 560 nm at the maximum concentration of Cu(II). A quantitative evaluation cannot be performed due to the difficulty in determining the molar absorption coefficient of the dye inside the material and to the limitation of the spectrophotometric analysis to the surface of the porous sample. By considering the molar absorption coefficient of RB in solution [14], only a rough estimation of the local concentration of the dye can be performed, yielding a µM range. Notably, the material which shows the highest sensitivity, with a reliable detection of Cu(II) at 1 ppm, is that composed of fibers obtained from DCM/acetone (Figure 7c and Figure S7c). On the basis of this result, we can envisage for this material a limit of detection of the order of 0.1 ppm. It can be noticed that a second absorption band at ca. 715 nm appears, upon Cu(II) exposure, in all cases and it is more pronounced in the softer fiber material (fibers from DCM/acetone, Figure 7c and Figure S7c). It can be attributed to an aggregated form of the formed RB.

Emission spectra have been recorded upon excitation at 320 nm (absorption maximum of RBH) and at 480 nm (selective excitation of the open form RB). The results are shown in Figures S8 and S9, as described in details below. Each spectrum is an average of two to four spectra taken in different areas of the solid materials, to reduce the effects of possible non-homogeneous distribution of the dye.

Excitation at 320 nm leads to prevalent excitation of RBH but also RB, formed upon reaction with Cu^2+^, is partially absorbing at this wavelength. The emission spectra thus present both emission bands of RBH and RB at 470 and 584 nm, respectively. The trend of the relative intensity of the band at 584 nm with respect to the one at 470 nm has been analyzed as a function of the copper concentration (Figure S8). An increase of the ratio with the concentration of the Cu(II) solution is observed up to 100 ppm, whereas at 200 ppm a decrease is occurring in all cases. This can be ascribed to the formation of aggregates of the produced RB that limit its emission output.

The spectra obtained upon excitation at 480 nm are shown in Figure S9. An intensification of RB emission intensity following the increase of the Cu(II) concentration is observed in all cases, with the exception of an unusual behavior for the fibers obtained from DCM/acetone in contact with a 100 ppm copper solution (Figure S9c). It can be noticed that an apparent red-shift of the emission maximum is observed with the increase of the copper concentration, and that in the samples in contact with the higher amount of Cu(II) an additional emission band at ca. 760 nm is appearing. The former effect is due to the large increase of RB absorption (Figure 7c and Figure S7c) that leads to a re-absorption effect in the region of emission at around 600 nm, while the latter feature can be ascribed to the formation of aggregates of RB on the material, when its concentration increases due to the reaction of RBH with Cu(II). Near-infrared (NIR) emission from RB aggregates, indeed, has been reported in the solid state or for high concentrated dyes entrapped into porous nanomaterials [24,26]. This outcome, already pointed out by the absorption data, is further confirmed by the excitation spectrum collected at 750 nm (Figure S10 for a selected case): an absorption profile different from that of RB (which is indeed observed upon collection at 620 nm) is obtained, with a significant contribution in the spectral region around 700 nm.

Emission lifetimes were measured for all the samples upon excitation at 331 nm, and the results are summarized in Table S1. The decays are multi-exponential in all cases, likely due to different local environments and distributions of the dyes. They can be treated with good approximation with a bi-exponential fitting procedure (Table S1). Decays collected at 470 nm, maximum of RBH emission, show a minor component with an average value of 2.4 ns and a major one (accounting for 50–70% of the decay) in the range 15–17 ns. Collection at 600 nm, emission maximum of the open form RB, leads to detect a lifetime of ca. 3.4 ns whose weight increases with the increase of the Cu(II) concentration and a longer lifetime of 10–14 ns, whose value is generally decreasing with the Cu(II) increment. The short lifetimes measured at both wavelengths are in good agreement with those detected in solution for RBH and RB (1.6 ns and 3.0 ns, respectively) while the longer components can be tentatively ascribed to aggregates or to re-absorption effects due to the high local concentration of the dyes [27,28]. The trend observed for relative amplitudes of the lifetimes measured at 600 nm confirm the occurrence of the recognition reaction in the materials.

Overall, the results point to a good response of the selected materials to Cu(II), with some drawbacks due to the formation of aggregates after the recognition reaction.

### 2.3. Fluorescence Confocal Laser Scanning Microscopy

**Figure 8:**
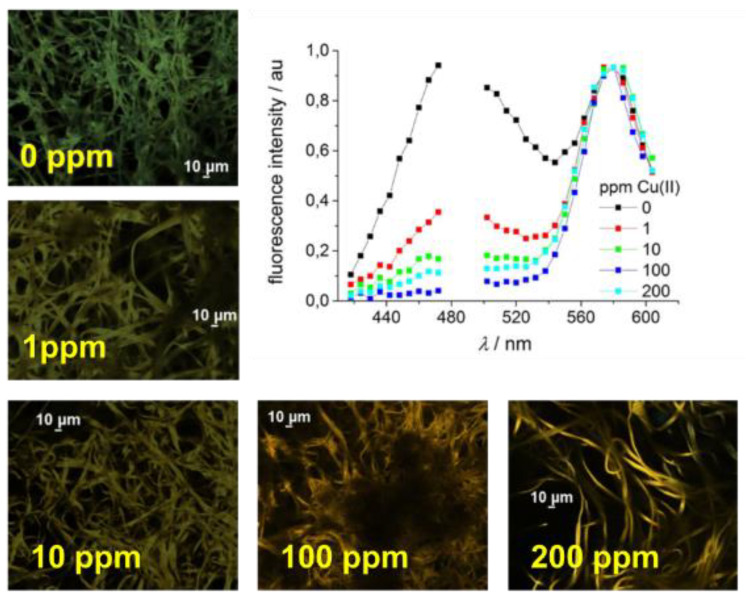
CLSM spectral images and graph with the confocal spectra obtained exciting at 405 nm for the fiber samples treated with different amounts of Cu(II) (due to a dichroic mirror reflecting light centered at 488 nm the spectra is not complete in the 480–500 nm range).

Fluorescence Confocal Laser Scanning Microscopy (CLSM) imaging of the materials loaded with 1% RBH, before and after reaction with different amounts of Cu(II), has been performed. The images show that the dye is well distributed in the porous membrane and fibers. Figure 8 shows the confocal images of fibers obtained from DCM/acetone treated and not with increasing amounts of Cu(II), and the corresponding spectra of selected regions of interest for excitation at 405 nm. The sample that has not been treated with Cu(II) displays a fluorescence spectrum similar to that of the closed RBH form. However, also in confocal imaging we discern a small presence of RB in the un-reacted material. In the treated samples the fluorescence spectra clearly show the appearance of the fluorescence of the open form (RB), peaking at ca. 580 nm, and the relative RB intensity follows the amount of Cu present in solution. In Figure S11, images of the Cu(II) treated fibers show that the spectra in different regions of interest are very similar throughout the whole sample. Also the images displaying the fluorescence intensity ratio I_(585 nm)_/I_(480 nm)_ with the color scale ranging from 0 to 3 confirm the trend of the spectra.

## 3. Materials and Methods

### 3.1. Materials

Cellulose acetate, dimethylformamide (DMF), dichloromethane (DCM), and acetone were from Sigma-Aldrich (St. Louis, MO, USA). Spectroscopic grade ethanol was from Carlo Erba (Barcelona, Spain). Rhodamine B (RB) was from Sigma-Aldrich (St. Louis, MO, USA) and Rhodamine B hydrazide (RBH) was prepared according to a procedure previously reported [14].

### 3.2. Film Preparation by Solution Casting

The cellulose acetate solution in acetone was gently poured in uniform petri dishes with diameter of 10 cm and the films left to dry at room temperature. A transparent non-porous film was produced, which was preserved under dry conditions in a desiccator at room temperature till further use [29].

### 3.3. Membrane Preparation by Phase Inversion

Flat cellulose acetate porous membranes were prepared by phase inversion technique. A suitable amount of cellulose acetate solution in DMF (20 wt.%) was poured on a glass plate and spread on the flat glass sheet surface by doctor knife. The produced uniform thin layer of cellulose acetate on the glass plate was dipped in deionized water smoothly. The formed oblique porous membrane was separated from water and preserved under dry conditions in a desiccator at room temperature till further use [30].

### 3.4. Fabrication of Nanofibers by Electrospinning

Cellulose acetate nanofibers were prepared by electrospinning using the following conditions: the polymer was dissolved in: (i) DMF and (ii) a DCM/acetone mixture in a concentration of 15% by weight and fed through a glass syringe attached with Teflon tube ended with a metallic needle, with a flow rate of 1 mL/h in case of using DCM/acetone mixture and 0.5 mL/h in case of using DMF. A potential difference of 16 and 21 KV was applied between the needle and the metal collector in case of using DMF/acetone mixture and DMF, respectively. The distance between the needle and the collector was kept at 15 cm [15,16,17].

### 3.5. RBH Pre-Loaded Fabrics

The RBH dye was loaded onto the cellulose acetate matrix by solution mixing (pre- loading). A pre-prepared DMF solution of RBH was used as dope solution to be mixed with the CA solution in different weight percentages. Pre-loaded fabrics were prepared by solution casting, phase inversion or electrospinning using the same conditions as for preparation for pure cellulose acetate fabrics.

### 3.6. RBH Post-Loaded Fabrics

RBH was loaded onto the CA fabrics (casted films, porous membranes and fibers) after their fabrication by adding, step by step, an ethanol solution of RBH into the fabricated materials (cut as 25 mm × 25 mm squares) and waiting for the complete evaporation of the solvent. Ethanol was chosen as an optimum solvent to allow a good solubilization of RBH without solubilizing the polymer.

The loading of RBH was performed on the basis of *w*/*w*% for the soft materials which could absorb completely the solution (phase deposition and fibers): two loadings have been tested: 0.5 and 1.0%. For the casted films, where the solution was not absorbed and dried on the top of the film, an average volume of solution with respect to the other samples was used (50 µL of a solution 0.978 mg/mL, which corresponds to 0.049 mg RBH on the surface for 0.5% loading, and double amount for 1% loading).

### 3.7. Morphological Characterization

Field Emission Scanning Electron Microscopy (FE-SEM) images of the top surface of the fabrics were obtained using Quanta FEG 250, FEI Co., The Netherlands.

### 3.8. Spectroscopic and Photophysical Characterization

All measurements were performed at room temperature. Spectroscopic grade ethanol was from Carlo Erba (Barcelona, Spain). Rhodamine B was from Sigma-Aldrich (St. Louis, MO, USA) and Rhodamine B hydrazide was prepared according to a procedure previously reported [14]. Absorption spectra of solutions were acquired with a Perkin-Elmer Lambda 650 UV/Vis spectrophotometer (Llantrisant, UK) in 1 mm optical path cuvettes. Reflectance spectra of solid samples were acquired with a Perkin–Elmer Lambda 950 UV/Vis/NIR spectrophotometer (Shelton, USA) equipped with a 100 mm integrating sphere. The samples were placed inside 25 mm × 25 mm quartz slides. For the post-loaded samples, the spectra were measured on the face loaded with RBH. The spectra obtained after contact with CuCl_2_ solutions were normalized on the maximum of absorption of the polymer (274 nm) to minimize the scattering effects.

Emission spectra of solutions were acquired in right-angle geometry with an Edinburgh FLS920 fluorimeter (Edinburgh, UK) equipped with a Peltier-cooled Hamamatsu R928 PMT (200–850 nm)and corrected for the wavelength dependent phototube response. Emission spectra of solid samples were collected with the same fluorimeter in front-face mode.

Fluorescence lifetimes were determined with an IBH 5000F time-correlated single-photon counting apparatus (Edinburgh, UK) by using pulsed NanoLED excitation sources at 331 and 465 nm. Analysis of the fluorescence decay profiles against time was accomplished with the Decay Analysis Software DAS6 provided by the manufacturer. The estimated error on lifetime determinations is 10%.

### 3.9. Fluorescence Confocal Laser Scanning Microscopy

Fluorescence imaging was performed on an inverted Nikon Ti-E microscope (Nikon Co., Shinjuku, Japan). The confocal microscope Nikon A1 is equipped with an Argon ion CW laser as well as a 405 and 485 nm pulsed/CW diode lasers (PicoQuant GmbH, Berlin, Germany). Images were collected using a Nikon Plan Apo VC 60X oil immersion objective with NA 1.40. Filters were set to register the fluorescence in the 460–500 nm, 500–550 nm, and 565–605 nm ranges. A Nikon A1 spectral module with a precisely corrected 32-channel multi-anode photomultiplier detector was used for spectral imaging. Wavelength resolution was set to 6 nm per anode. Spectral images were obtained by exciting the sample at 488 nm in CW mode. A dichroic mirror reflecting 405 nm and 488 nm excitation light was used during confocal imaging.

### 3.10. Overview of the Studied Samples and Processes

Table 1 summarizes the types of cellulose acetate materials studied, their preparation conditions, their loading with RBH and the parameters adopted for Cu(II) sensing.

## 4. Conclusions

Rhodamine B hydrazide was successfully loaded into cellulose acetate, a natural polymer derivative. Among the prepared samples, the most suitable fabrics for RBH loading were the porous phase-deposited membrane and two types of nanofibers, differing in the fiber diameter.

The post-treatment deposition, where the dye is loaded after the preparation of the polymeric material, ensured good stability with respect to degradation and the dye retained the optical properties observed in solution.

Optimized RBH loading occurred at 1% (*w*/*w*) for both the porous membrane and the nanofibers. The selected samples were then tested for Cu(II) sensing affording a satisfactory response, especially in terms of colorimetric detection. Nanofibers electrospun from DCM/acetone and loaded with RBH were found to be the most sensitive fabric towards Cu(II). Further studies on the sensitivity and selectivity of this composite material are under way. These promising results open the way to the development of bio-compatible and ready-to-use sensing systems.

## Figures and Tables

**Table 1:** Summary of the sample preparation procedures, dye loading, and Cu(II) sensing.

Sample	Solvent(s) Used for Preparation	Preparation Procedure	Loading with RBH (*w/w*%) ^a^	Immersion Time with Cu(II) Solution	pH of the Cu(II) Solution	Cu(II) Concentration
Casted film	Acetone	Casting	0.50%	-	-	-
			1.00%	-	-	-
Membrane	Acetone/DMF	Phase inversion	0.50%	-	-	-
			1.00%	1 night	7	1, 10, 100, and 200 ppm
Nanofibers	DMF	Electrospinning	0.50%	-	-	-
			1.00%	1 night	7	1, 10, 100, and 200 ppm
Nanofibers	DCM/Acetone	Electrospinning	0.50%	-	-	-
			1.00%	1 night	7	1, 10, 100, and 200 ppm

^a^ Post-loading procedure.

## Supplementary Materials

The following are available online, Figure S1: Arbitrarily scaled absorption (full line, ε/M^–1^ cm^−1^: 59,400 at 240 nm, 36,500 at 273 nm, and 13,300 at 312 nm) and emission (dashed line) spectra of RBH in ethanol at room temperature; Figure S2: Arbitrarily scaled absorption (full line) and emission (dashed line) spectra of RB in ethanol at room temperature; Figure S3. Absorption spectra of samples with 0, 0.5, and 1% (*w/w*) post-loading of RBH: (a) casted film, (b) phase deposited membrane, (c) fibers from DMF, and(d) fibers from DCM/acetone; Figure S4: Corrected emission spectra of samples with 0, 0.5, and 1% (*w/w*) post-loading of RBH: (a) casted film, (b) phase deposited membrane, (c) fibers from DMF, and d) fibers from DCM/acetone. Excitation at 320 nm; Figure S5: Normalized corrected excitation spectra, collected at 430 nm, for fibers from DMF (full line) and fibers from DCM/acetone (dashed line); Figure S6: Corrected excitation spectra, collected at 500 and 600 nm, for phase deposited membrane sample loaded with 1% RBH; Figure S7. Absorption spectra of the RBH loaded polymeric materials, prior and after 1 night immersion in CuCl_2_ water solutions (pH = 7) at different Cu(II) concentrations: (a) porous membrane, (b) fibers from DMF, and (c) fibers from DCM/acetone. In the insets, a magnification of the 400–780 nm region is reported; Figure S8: Ratios of emission intensity at 584 vs. 470 nm, as a function of Cu(II) concentration, collected upon excitation at 320 nm for: phase deposited membrane (black), fibers from DMF (red), fibers from DCM/acetone (blue), loaded with 1% RBH, after contact with CuCl_2_ water solutions (pH = 7); Figure S9: Corrected emission spectra, collected upon excitation at 480 nm, of the materials loaded with 1% RBH after contact with CuCl_2_ water solutions (pH = 7) at different Cu(II) concentrations: (a) phase deposited membrane, (b) fibers from DMF, and (c) fibers from DCM/acetone. In (a) the purple and pink spectra have been divided by 5; Figure S10: Arbitrarily scaled corrected excitation spectra, collected at 620 nm and 750 nm, for fibers from DCM/acetone loaded with 1% RBH after contact with 200 ppm CuCl_2_ water solution (pH = 7); Table S1: Lifetimes measured on solid materials, upon excitation at 331 nm, at the indicated wavelengths; Figure S11: The graphs show six normalized confocal fluorescence spectra of various regions of interest collected from each spectral image below. The images at the bottom represent the fluorescence intensity ratio I_585 nm_/I_480 nm_ ranging from 0,1 (purple) to 3 (red).

## Notes

^§^ The reflectance spectrum of the bare polymeric material in the 230–320 nm range has been omitted because higher than 100%, probably due to scattering effects of the porous membrane.
