# Supplementary material for: Cellulose Acetate Fabrics Loaded with Rhodamine B Hydrazide for Optical Detection of Cu(II)
Source: Molecules. 2020 Aug 17;25(16):3751. doi: 10.3390/molecules25163751 (PMC7464765; doi:10.3390/molecules25163751)
Supplement: Supplementary file 1 [file molecules-25-03751-s001.pdf]

Supporting Information

for

Cellulose Acetate Fabrics Loaded with Rhodamine B Hydrazide for Optical Detection of Cu(II)

Rania E. Morsi,[a,b*] *Moataz Elsawy,*[a] *Ilse Manet,*[b] *Barbara Ventura*[b*]

[a] Egyptian Petroleum Research Institute, Nasr City, PO Box 11727, Cairo, Egypt.

[b] Istituto per la Sintesi Organica e la Fotoreattività (ISOF), Consiglio Nazionale delle Ricerche (CNR), Via P. Gobetti 101, 40129 Bologna, Italy.

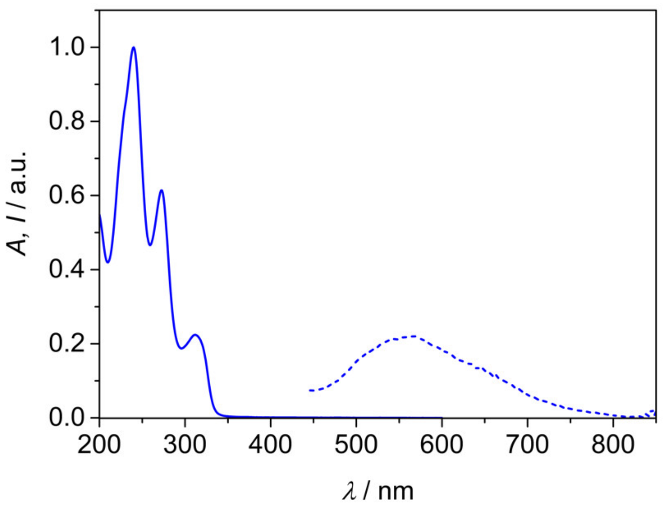

Figure S1. Arbitrarily scaled absorption (full line, ε / M^{-1} cm^{-1}: 59.400 at 240 nm, 36.500 at 273 nm, and 13.300 at 312 nm) and emission (dashed line) spectra of RBH in ethanol at room temperature.

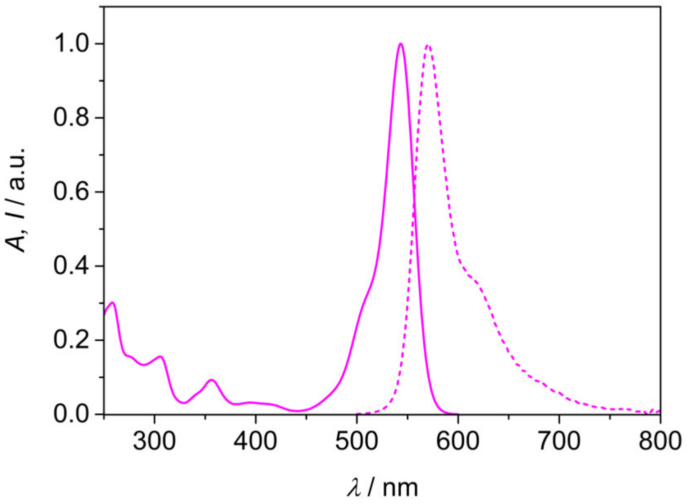

Figure S2. Arbitrarily scaled absorption (full line) and emission (dashed line) spectra of RB in ethanol at room temperature.

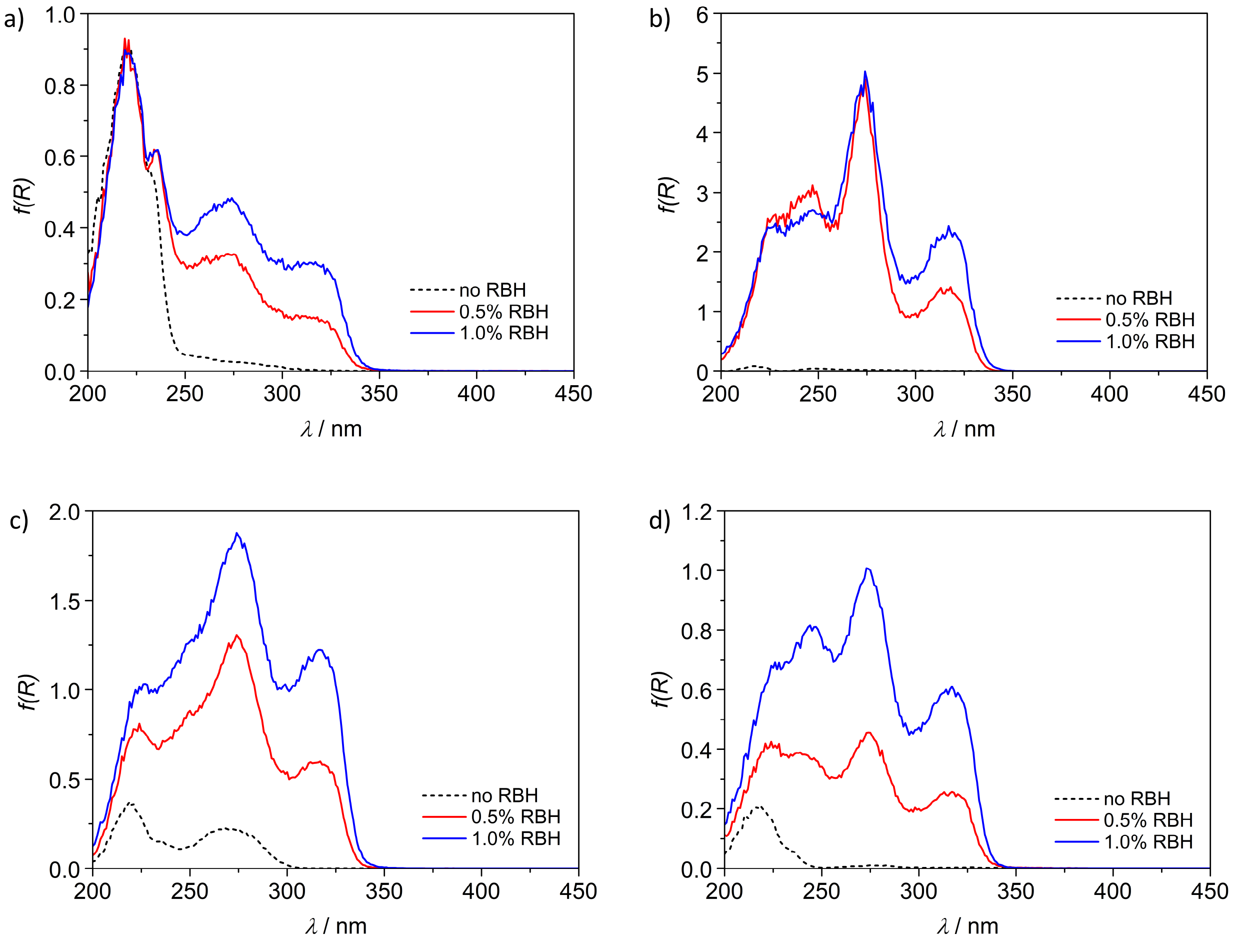

Figure S3. Absorption spectra of samples with 0, 0.5, and 1% (w/w) post-loading of RBH: (a) casted film, (b) phase deposited membrane, (c) fibers from DMF, and (d) fibers from DCM/acetone.

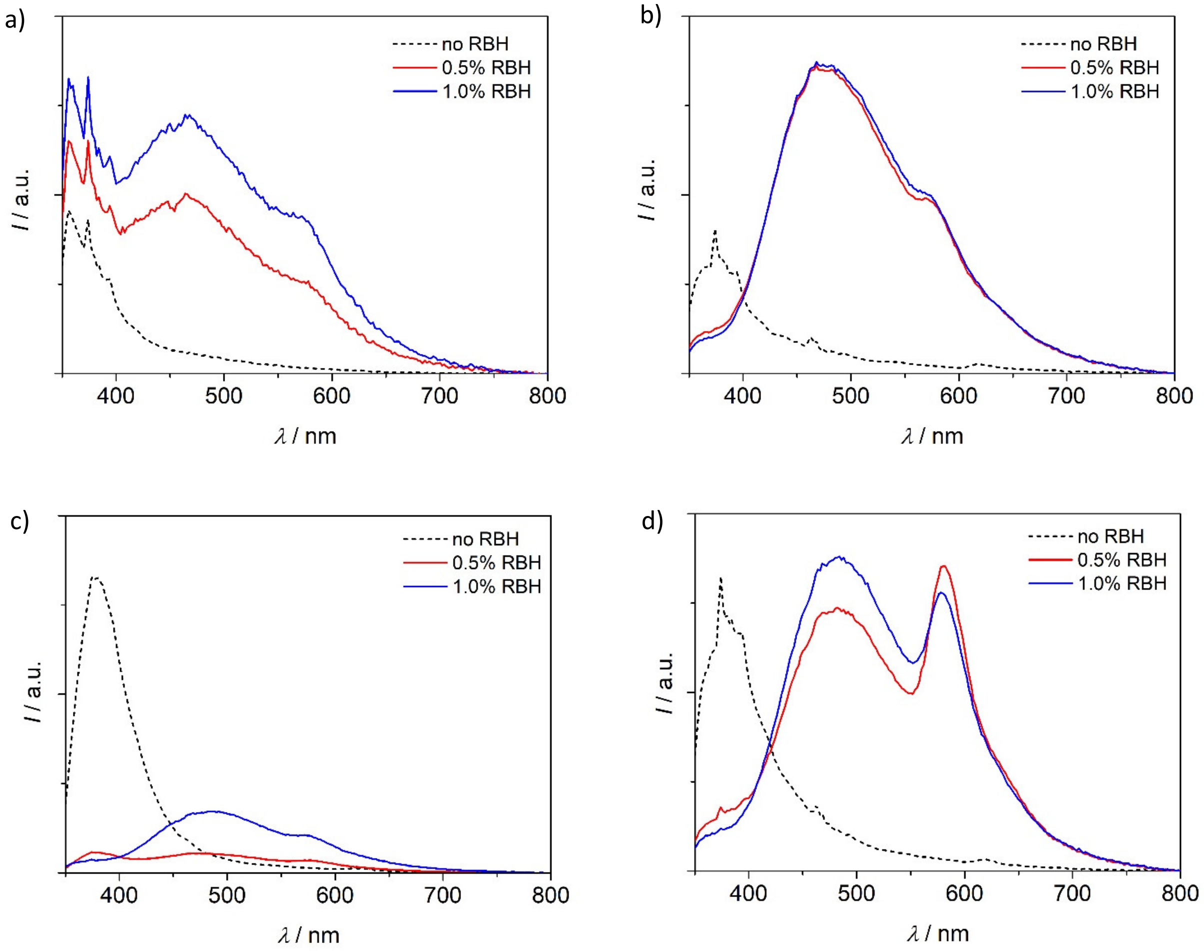

Figure S4. Corrected emission spectra of samples with 0, 0.5, and 1% (w/w) post-loading of RBH: a) casted film, b) phase deposited membrane, c) fibers from DMF, and d) fibers from DCM/acetone. Excitation at 320 nm.

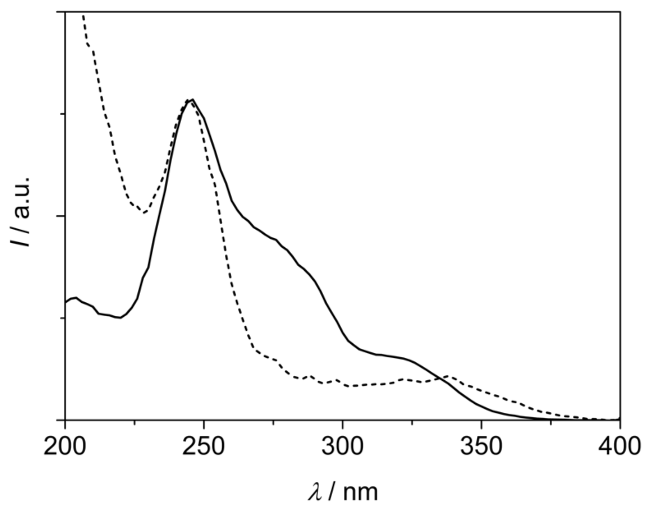

Figure S5. Normalized corrected excitation spectra, collected at 430 nm, for fibers from DMF (full line) and fibers from DCM/acetone (dashed line).

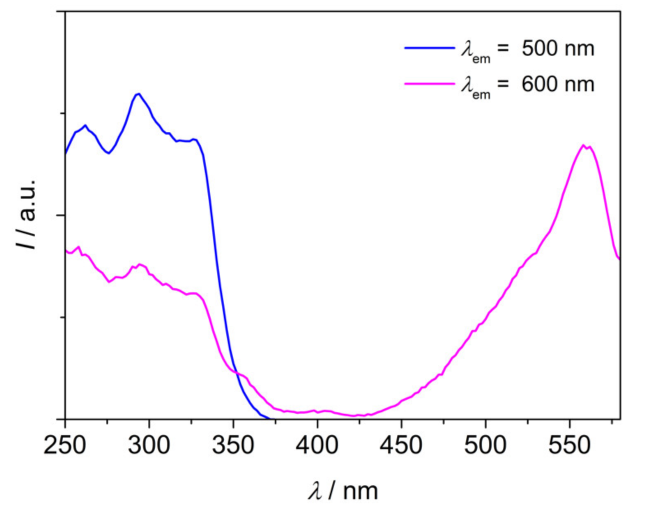

Figure S6. Corrected excitation spectra, collected at 500 nm and 600 nm, for phase deposited membrane sample loaded with 1% RBH.

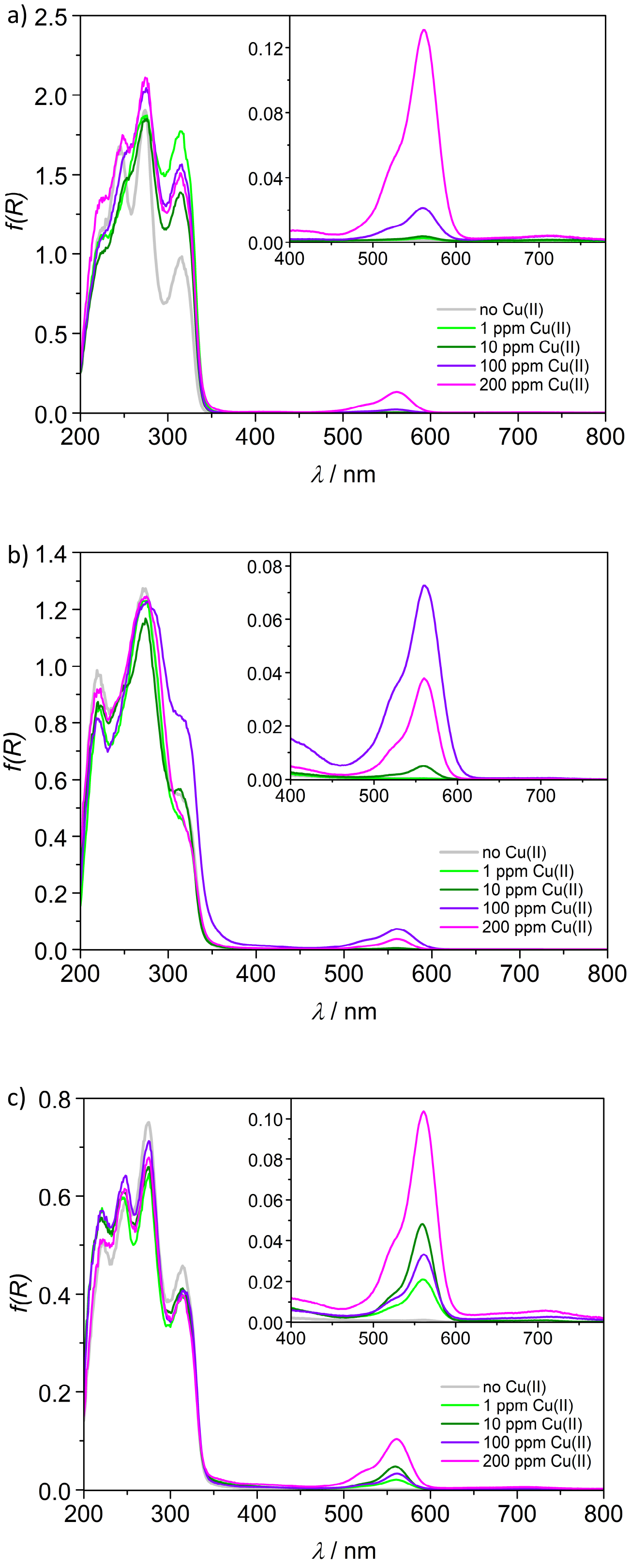

Figure S7. Absorption spectra of the RBH loaded polymeric materials, prior and after 1 night immersion in $CuCl_2$ water solutions (pH = 7) at different Cu(II) concentrations: (a) porous membrane, (b) fibers from DMF, and (c) fibers from DCM/acetone. In the insets, a magnification of the 400-780 nm region is reported.

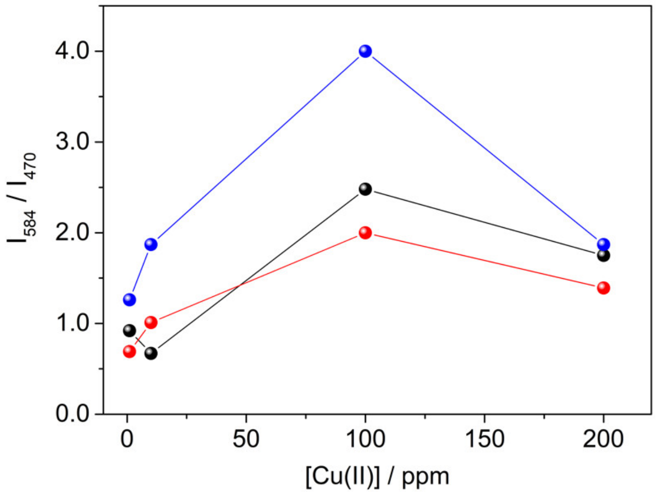

Figure S8. Ratios of emission intensity at 584 vs. 470 nm, as a function of Cu(II) concentration, collected upon excitation at 320 nm for: phase deposited membrane (black), fibers from DMF (red), fibers from DCM/acetone (blue), loaded with 1% RBH, after contact with $CuCl_2$ water solutions (pH = 7).

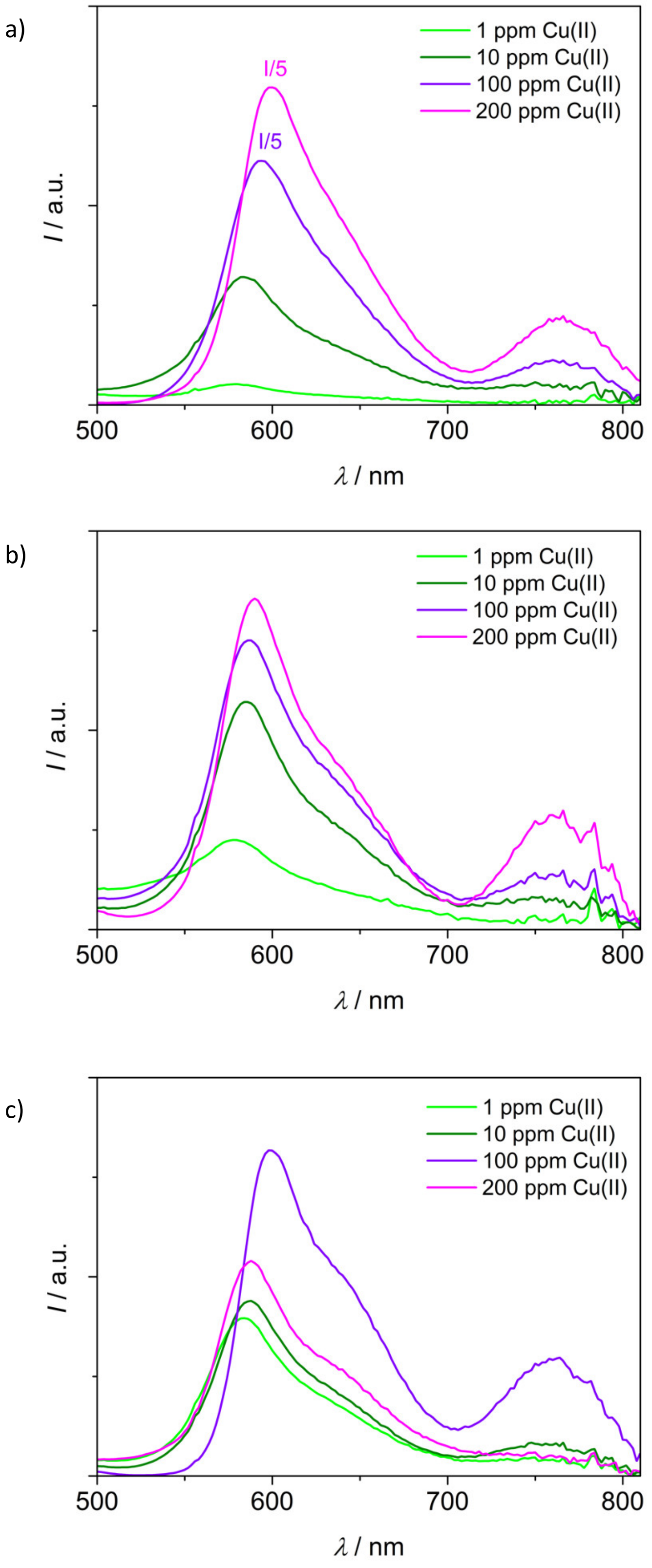

Figure S9. Corrected emission spectra, collected upon excitation at 480 nm, of the materials loaded with 1% RBH after contact with $CuCl_2$ water solutions (pH = 7) at different Cu(II) concentrations: a) phase deposited membrane, b) fibers from DMF, and c) fibers from DCM/acetone. In a) the purple and pink spectra have been divided by 5.

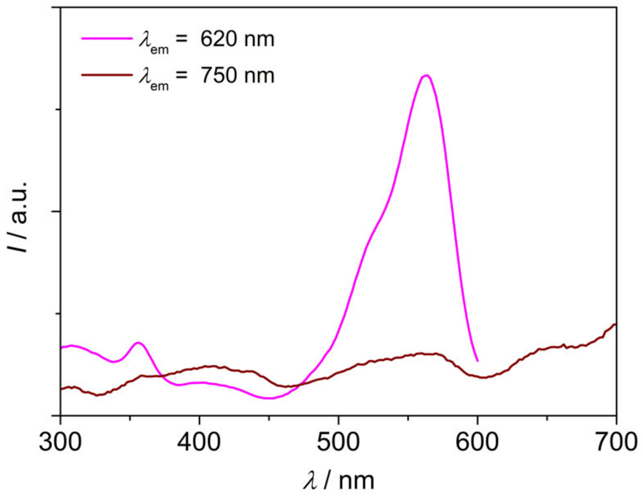

Figure S10. Arbitrarily scaled corrected excitation spectra, collected at 620 nm and 750 nm, for fibers from DCM/acetone loaded with 1% RBH after contact with 200 ppm $CuCl_2$ water solution (pH=7).

Table S1. Lifetimes measured on solid materials, upon excitation at 331 nm, at the indicated wavelengths.[a]

	Phase deposited membrane		Fibers from DMF		Fibers from DCM/acetone	
	τ / ns		τ / ns		τ / ns	
	λ_{em}= 470 nm	λ_{em}= 600 nm	λ_{em}= 470 nm	λ_{em}= 600 nm	λ_{em}= 470 nm	λ_{em}= 600 nm
No Cu(II)	1.9 (24%)<br>15.3 (76%)	-	3.1 (36%)<br>18.6 (64%)	-	2.7 (30%)<br>16.0 (70%)	-
1 ppm Cu(II)	2.8 (33%)<br>17.6 (67%)	3.7 (50%)<br>14.7 (50%)	2.5 (38%)<br>17.6 (62%)	2.6 (35%)<br>13.7 (65%)	1.8 (32%)<br>15.2 (68%)	3.6 (66%)<br>12.5 (34%)
10 ppm Cu(II)	2.7 (30%)<br>16.6 (70%)	4.0 (54%)<br>14.3 (46%)	2.4 (33%)<br>16.6 (67%)	3.2 (54%)<br>14.1 (46%)	2.1 (31%)<br>15.0 (69%)	3.5 (67%)<br>10.7 (33%)
100 ppm Cu(II)	2.6 (30%)<br>16.5 (70%)	4.0 (56%)<br>12.7 (44%)	2.8 (40%)<br>17.9 (60%)	2.7 (65%)<br>9.8 (35%)	1.8 (36%)<br>14.2 (64%)	3.2 (70%)<br>10.0 (30%)
200 ppm Cu(II)	2.0 (34%)<br>16.0 (66%)	2.5 (57%)<br>8.3 (43%)	2.3 (43%)<br>17.7 (57%)	2.1 (67%)<br>7.8 (33%)	2.3 (34%)<br>15.3 (66%)	3.2 (64%)<br>10.2 (36%)

[a]A bi-exponential fitting has been applied in all cases and the relative amplitudes are indicated in parenthesis.

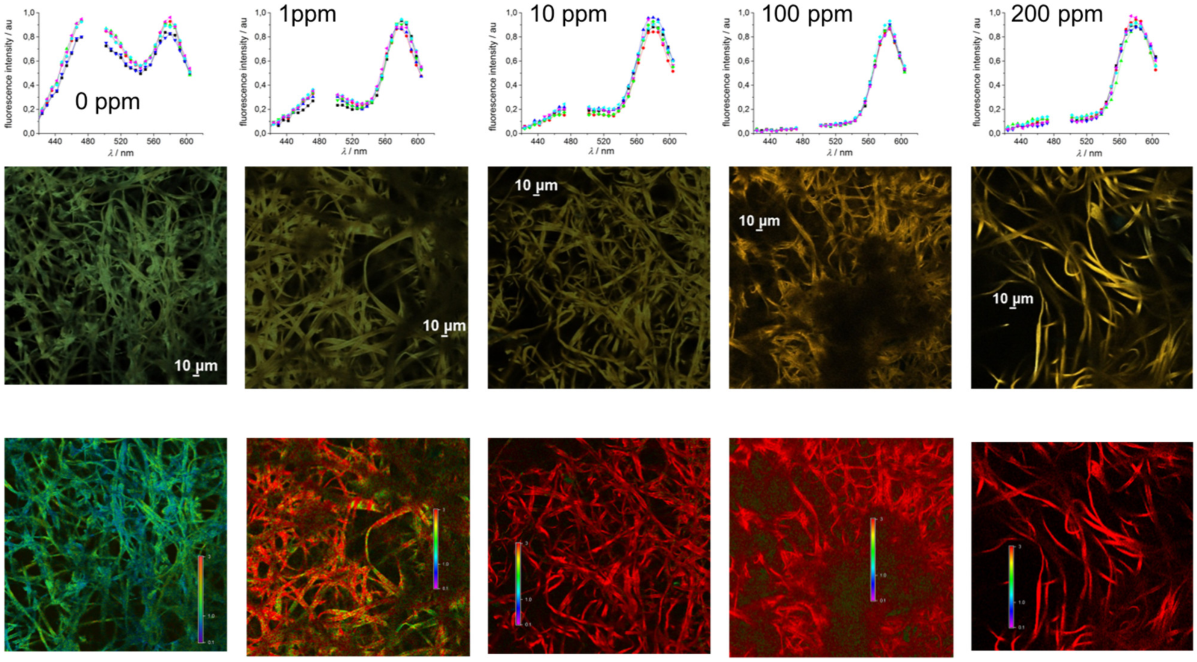

Figure S11. The graphs show six normalized confocal fluorescence spectra of various regions of interest collected from each spectral image below. The images at the bottom represent the fluorescence intensity ratio $I_{585\ nm}$ / $I_{480\ nm}$ ranging from 0,1 (purple) to 3 (red).
